# Scaffold protein RhCASPL1D1 stabilizes RhPIP2 aquaporins and promotes flower recovery after dehydration in rose (*Rosa hybrida*)

**DOI:** 10.1093/hr/uhaf119

**Published:** 2025-04-30

**Authors:** Kun Liu, Tao Zhang, Siqi Zhao, Jin Chen, Wentong Zhou, Siyu Chen, Yubi Su, Qinglin Liu, Junping Gao, Changqing Zhang

**Affiliations:** Department of Ornamental Horticulture, College of Horticulture, China Agricultural University, Beijing 100193, China; Department of Ornamental Horticulture, College of Horticulture, China Agricultural University, Beijing 100193, China; Department of Ornamental Horticulture, College of Horticulture, China Agricultural University, Beijing 100193, China; Department of Ornamental Horticulture, College of Horticulture, China Agricultural University, Beijing 100193, China; Department of Ornamental Horticulture, College of Horticulture, China Agricultural University, Beijing 100193, China; Department of Ornamental Horticulture, College of Horticulture, China Agricultural University, Beijing 100193, China; Department of Ornamental Horticulture, College of Horticulture, China Agricultural University, Beijing 100193, China; Department of Ornamental Horticulture, College of Horticulture, China Agricultural University, Beijing 100193, China; Department of Ornamental Horticulture, College of Horticulture, China Agricultural University, Beijing 100193, China; Department of Ornamental Horticulture, College of Horticulture, China Agricultural University, Beijing 100193, China

## Abstract

Water deficit during flowering can lead to petal wilting, necrosis, and sterility, severely limiting crop fertilization and yield. Therefore, rapid recovery of floral organs after dehydration is essential for angiosperms to achieve their full reproductive potential. Aquaporins (AQPs) are bidirectional membrane channels mediating water transmembrane transport. Plasma membrane intrinsic proteins (PIPs), one of AQP subfamily, play a key role in flower opening and dehydration responses. However, it still needs to be elucidated how PIPs are involved in flower recovery after stress. Cut rose (*Rosa hybrida*), a globally important ornamental flower, undergoes dehydration and rehydration during the postharvest process. Here, we show that the scaffold protein-encoding gene *CASP-LIKE PROTEIN 1D1* (*RhCASPL1D1*), expressed during flower opening and dehydration, promotes flower recovery after dehydration. Silencing *RhCASPL1D1* in rose petals and calli hindered cell recovery following dehydration and reduced the rate of water uptake, whereas *RhCASPL1D1* overexpression had the opposite effect. Ethylene upregulated *RhCASPL1D1* expression, and RhCASPL1D1 physically interacted with RhPIP2s at the plasma membrane. This interaction facilitated RhPIP2s retention to delay its degradation at the plasma membrane and enhanced proteins abundance under dehydration stress. Taken together, our findings reveal a potential mechanism involved in RhCASPL1D1 scaffold regulating flower recovery after dehydration stress.

## Introduction

Plants continuously face various stresses, among which water deficit is a universal abiotic stress that dramatically limits plant growth and development [1]. Flowering represents the pivotal transitional period of plants from vegetative to reproductive growth. When plants experience water deficit during flowering, flowers must recover rapidly to ensure reproductive success. Thus, plants have evolved diverse and complex strategies to balance dehydration responses and flowering, including drought avoidance and drought escape [2, 3]. Although the drought response in flowers has been well documented, the underlying mechanisms by which floral organs rapidly and fully recover following water deficit remain elusive.

Aquaporins (AQPs) function to maintain water balance, which is important for plant adaptation to drought stress [4]. Plasma membrane (PM) intrinsic proteins (PIPs) are the most abundant AQPs and are responsible for water transport across the PM. Based on their amino acid sequence, PIPs are classified as either PIP1 or PIP2 subgroups. Compared to PIP1 members, PIP2s have higher activities to transport water in plants drought responses [5]. Given that PIPs are bidirectional water transport channels, PIPs abundance and activity in the PM must be precisely regulated to maintain water homeostasis under water deficit conditions.

PIP2 abundance in the PM is transcriptionally and post-translationally downregulated under stress, enhancing the water retention capacity of plants. For example, the plant A/T-rich sequence and zinc-binding protein PLATZ4 transcriptionally regulates *AtPIP2;8* expressions to limit water loss from stomata in Arabidopsis [6]. Moreover, several protein degradation-related enzymes, such as the E3 ligase RING membrane-anchor 1 homolog 1 (RMA1H1), ubiquitin-conjugating enzyme 32 (UBC32), and the dehydrin cold acclimation-specific 31 (CAS31), mediate PIP2s degradation through the ubiquitin-proteasome system or via autophagy and therefore enhance crop drought tolerance [7, 8]. Lastly, stress-induced H_2_O_2_ accumulation triggers AtPIP2;1 and AtPIP2;7 internalization from the PM to modify root cell water conductivity [9].

Interestingly, increasing evidences indicate that PIPs abundance at the PM remains unchanged or even increases in some crop species and plant organs, which is likely a strategy for plants to withstand and recover from multiple abiotic stresses. Under short-term salt stress, *AtPIP2;7* expression decreased, while its protein abundance was unaffected [10]. In the halophyte *Mesembryanthemum crystallinum*, a transcription-independent increase in PIPs abundance at the PM triggered by NaCl is an adaptative process to osmotic stress [11]. Temperature-induced GsPIP2;2 accumulation in the PM regulates flower re-opening from cool in *Gentiana scabra* [12]. In above processes, the phytohormone ethylene is thought to regulate PIP abundance and activity on the PM [13, 14], but the underlying mechanisms remain poorly understood.

Scaffold proteins are important accessory players in cells that recruit signaling molecules, bringing about considerable changes in client protein enzymatic activity, localization and stability, and interactions with other proteins [15, 16]. Casparian strip membrane domain proteins (CASPs) are specifically expressed in the Casparian strip (CS) and contribute to its formation [17]. These proteins are highly stable in their membrane domain, exhibiting all the characteristics of a membrane scaffold. CASP-like proteins (CASPLs) share a domain (PF04535) in common with CASPs and have tissue-specific expression patterns in various cell types in addition to the endodermis [18]. Moreover, bioinformatics analysis revealed that in patchouli (*Pogostemon cablin*) the cis-regulatory elements of different *PatCASPLs* within the same subcluster vary, suggesting that *CASPLs* may have distinct functions [19].

CASPL family genes play an important role in numerous biological processes, e.g. restricting pathogen infections [20], altering cold tolerance [21], and regulating wax synthesis under drought stress [22]. A previous study revealed that AtCASPL1D1 and AtCASPL1D2 interact with AtPIP2;1, although the biochemical effects of this interaction are unclear, suggesting a potential role of CASPL1Ds in water transport under water deficit [23]. Nevertheless, the understanding of the molecular mechanisms involving CASPLs is very limited.

Cut rose (*Rosa hybrida*), an important ornamental flower, undergoes dehydration and rehydration during postharvest handling. During the production and postharvest stages, drought and dehydration are unavoidable stresses resulting in failure to normally open, which severely compromises product quality and quantity [24, 25]. For rose flowers, the ability to quickly and fully recover after water deficit is an important quality trait. While much research has focused on flower dehydration tolerance, relatively little is known regarding the mechanisms by which flowers recover during rehydration, which is crucial as it determines final ornamental values. Here, we identified *RhCASPL1D1,* which was strongly induced by ethylene in petals during dehydration and rehydration. RhCASPL1D1 localizes to the PM and acts as scaffold for several RhPIP2s and enhances their stability under dehydration stress. The interaction between RhCASPL1D1 and RhPIP2s facilitates PIP2s retention at the PM and thus enables the rapid recovery of flowers after dehydration stress.

## Results

### Genome-wide identification of dehydration- and aging-induced *CASPL* genes in rose flowers

**Figure 1:**
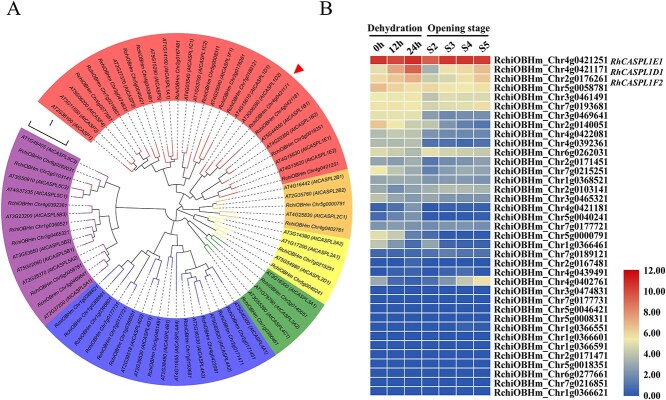
Phylogenetic analysis and expression of *RhCASP-like* family genes. **A)** Phylogenetic analysis of *CASPL* family genes in *Rosa chinensis* and Arabidopsis. The phylogenetic tree was constructed using MEGA11 with 1000 bootstrap replicates. *CASPLs* were divided into six clusters. The scale bar indicates evolutionary distance. *RhCASPL1D1* is indicated by an arrowhead. **B)** Heatmap of *RhCASP-like* family genes expression patterns during flower opening and dehydration. Transcriptional levels in the heatmap are log_2_-based FPKM values from RNA-seq in the rose cultivar ‘Samantha’. S2-S5, flowers at opening stage 2 to stage 5.

CASPLs are members of the eukaryotic MARVEL protein superfamily. They are found in all major divisions of land plants and perform diverse biological functions [19, 26]. To investigate whether *RhCASPLs* are involved in dehydration response in rose flowers, *RhCASPL* family genes were identified at the genome-wide level. Based on homology with the 39 CASPL protein sequences of Arabidopsis (*Arabidopsis thaliana*), we identified 37 RhCASPL family members in the rose genome (https://lipm-browsers.toulouse.inra.fr/pub/RchiOBHm-V2). The conserved CASPL (PF04535) and MARVEL (PF01284) domains, which are characteristic of the CASPL protein family, were verified using HMMER [27]. Chromosome mapping revealed that the *RhCASPLs* are unevenly distributed within the rose genome (Fig. S1). To explore the phylogenetic relationships within the *RhCASPL* gene family, we reconstructed the phylogeny of 76 CASPL protein sequences from *Rosa chinensis* and Arabidopsis using MEGA11 [28]. The results showed that RhCASPL members could be divided into six subclusters, which is consistent with the evolutionary relationship among CASPLs in Arabidopsis (Fig. 1A) [21].

**Figure 2:**
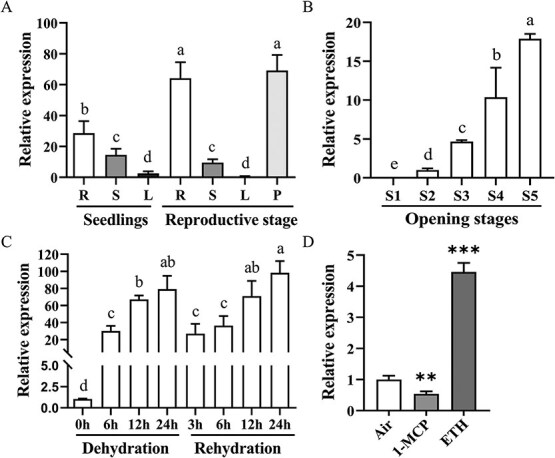
*RhCASPL1D1* expression is upregulated during flower opening and by osmotic stress and ethylene. RT-qPCR analysis of *RhCASPL1D1* expression in different rose organs **A)**, during different flower opening stages **B)**, and during dehydration and rehydration **C)**. R, root; S, stem; L, leaf; P, expanded flower petal; S1-S5, flower opening stage 1 to stage 5. Different lowercase letters indicate significant differences (*P* < 0.05; one-way ANOVA, followed by Tukey’s HSD test). **D)** Relative expression of *RhCASPL1D1* under ethylene by RT–qPCR. Flowers in opening stage 2 were exposed 10 μL L^−1^ ethylene or 2 μL L^−1^ 1-MCP for 24 h, respectively, with air as a control. The error bars are ± SD. Statistical significance was calculated by Student’s *t*-test; ^**^ P < 0.01, and ^***^ P < 0.001.

**Figure 3:**
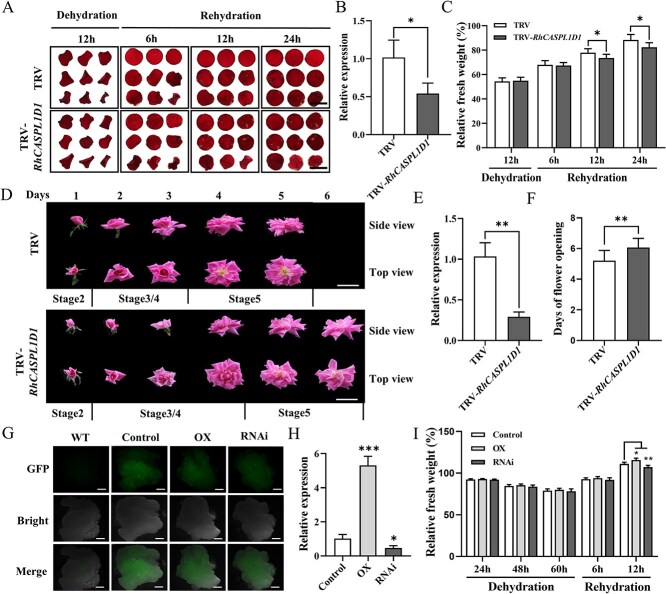
*RhCASPL1D1* promotes flower opening and tissue recovery during rehydration. **A)** Phenotypes of *RhCASPL1D1*-silenced petal discs during dehydration and rehydration. *RhCASPL1D1*-silenced (TRV*-RhCASPL1D1*) and control (TRV) petal discs were subjected to 12-h dehydration followed by 24-h rehydration. Scale bar, 1 cm. **B)**  *RhCASPL1D1* expression level in TRV and TRV*-RhCASPL1D1* petal discs. **C)** Relative fresh weight of *RhCASPL1D1*-silenced petal discs. Percentage values are ratios of fresh weights measured at the indicated time points to the initial weights. Data are means ± SD, *n* = 9. **D)** Flower opening phenotypes of the TRV control and *RhCASPL1D1*-silenced rose plantlets at different stages. Scale bar, 5 cm. **E)** Relative expression levels of *RhCASPL1D1* in TRV control and *RhCASPL1D1*-silenced flower petals. Petals of flowers in opening stage 5 were used for RT-qPCR analysis. Data are means ± SD, *n* = 18. **F)** Duration of the opening phase of RhCASPL1D1-silenced rose flowers. The number of days between stage 2 to stage 5 of flower opening were counted. Values are means ± SD (*n* = 18). **G)** Confocal micrographs of transgenic rose calli. GFP fluorescence was observed via a laser scanning confocal microscope (Zeiss LSM 800) under 488 nm wavelength. Scale bars, 2 mm. **H)** RT-qPCR analysis of RhCASPL1D1 expression in transgenic rose calli. Each value is the mean of three replicates ± SD. **I)** Relative fresh weight of transgenic rose calli. Transgenic calli was exposed to air for 60 h (dehydration) and then transferred to medium with 3% agar for recovery (rehydration). Relative fresh weight was determined at the indicated time points. Values are shown as means ± SD (*n* = 6). WT, non-transformed calli; OX, *RhCASPL1D1*-OX calli; RNAi, *RhCASPL1D1*-RNAi calli. All statistically significant differences are indicated by asterisks (^*^P < 0.05, ^**^P < 0.01; Student’s *t*-test).

To identify candidate dehydration- and aging- responsive *RhCASPL* genes in rose flower, we analyzed our previous transcriptomic data of rose genes differentially regulated during 0–24 h dehydration and flower opening processes with stages 2–5 defined by Ma *et al.* [29]. Cluster heatmap of 37 *RhCASPL* transcripts was constructed using the fragments per kilobase of exon per million mapped reads (FPKM) values (Fig. 1B; Table S1). We found that two *RhCASP-like1* cluster members, *RhCASPL1D1* and *RhCASPL1F2,* were obviously up-regulated during dehydration treatments and flower opening processes, and *RhCASPL1E1* showed relatively higher expression level at stages 3 and 4. *RhCASPL1D1* with the most significant expression change was selected for further study.

To characterize the protein structure and properties of RhCASPL1D1, we performed a phylogenetic analysis and multiple sequence alignment of RhCASPL1D1 and its putative homologs from other plant species. Protein pairwise similarity matrix revealed that RhCASPL1D1 shares highly sequence similarity (82.49% to 98.54%) with selected homologs (Table S2). Phylogenetic analysis indicated that RhCASPL1D1 was most similar to MdCASPL1D1 from apple (*Malus domestica*) (Fig. S2A). Multiple sequence alignment analysis revealed that RhCASPL1D1 and its orthologous contain a highly conserved CASPL (PF04535) domain. RhCASPL1D1 has four predicted transmembrane domains, with cytoplasmic N and C termini and a short intracellular loop (Fig. S2B and C). Instability index is used to predict *in vivo* protein stability dependent on protein primary structures [30]. According to ProtParam program (https://web.expasy.org/protparam/), a protein whose instability index is smaller than 40 is predicted as stable one. The instability index of RhCASPL1D1 is 23.79, indicating RhCASPL1D1 stability as scaffold proteins. Protein characterization suggests that RhCASPL1D1 may function as a membrane scaffold protein to interact with potentially binding partners.

### Dehydration-induced *RhCASPL1D1* expression is ethylene-dependent

To elucidate where RhCASPL1D1 functions, the transcription profile of *RhCASPL1D1* was detected via reverse transcription quantitative PCR (RT-qPCR) in rose different organs. RT-qPCR analysis revealed that *RhCASPL1D1* expression exhibits high tissue specificity both in rose young seedlings and flowering plantlets: it is highly expressed in open flowers and roots, but was barely detected in leaves (Fig. 2A).

We then investigated *RhCASPL1D1* expression in flowers at different opening stages and flowers subjected to dehydration stress. In terms of flower opening, *RhCASPL1D1* expression increased 20-fold at stage 5 compared to stage 2 (Fig. 2B). In response to a 6-h dehydration treatment, *RhCASPL1D1* was rapidly induced in petals, exhibiting a 30-fold increase. After three hours of rehydration, *RhCASPL1D1* expression slightly decreased before increasing again (Fig. 2C). These results suggest that *RhCASPL1D1* may function in the flower dehydration response and in the recovery of flower opening during subsequent rehydration. In addition, we tested how different phytohormones regulate *RhCASPL1D1* expression. *RhCASPL1D1* was strongly induced in petals by ethylene treatment. In contrast, 1-methylcyclopropene (1-MCP), a competitive inhibitor of ethylene receptors, inhibited *RhCASPL1D1* expression (Fig. 2D). These results confirmed that the dehydration stress-induced expression of *RhCASPL1D1* is dependent on ethylene.

### RhCASPL1D1 promotes rapid tissue recovery after dehydration

To shed light on the potential role of RhCASPL1D1 in flower opening and dehydration responses, we silenced *RhCASPL1D1* in rose petal discs and plantlets (TRV*-RhCASPL1D1*) through virus-induced gene silencing (VIGS) via agroinfiltration. RT-qPCR analysis showed that the *RhCASPL1D1* transcript abundance in TRV*-RhCASPL1D1* samples were significantly reduced (Fig. 3B and E). Infiltrated petal discs were dehydrated for 12 h followed by 24-h rehydration (Fig. 3A). In the dehydration phase, no obvious differences were observed in the fresh weight of all transformed petal discs. However, compared to TRV control, significantly lower relative fresh weights were observed at 12 and 24 h of rehydration in the *RhCASPL1D1*-silenced rose petal discs (Fig. 3C). As for the *RhCASPL1D1*-silenced plantlets, we observed delayed petal spreading (stages 2–5) of flowers (6.0 days), compared to control (5.2 days) (Fig. 3D and F). To validate the VIGS results, we generated *RhCASPL1D1*-*GFP* overexpressing petal discs by agroinfiltration, using the empty pSuper-*GFP* vector as the negative control (Fig. S3A). Increased *RhCASPL1D1* transcript levels in the overexpressing line were confirmed by RT-qPCR (Fig. S3B). Twenty-four hours after rehydration, the relative fresh weight of *RhCASPL1D1*-*GFP* petal discs were 95.9%, compared to 88.0% in the negative control (Fig. S3C). These results imply that *RhCASPL1D1* promotes petal recovery after dehydration and flower opening in rose.

To better understand *RhCASPL1D1* function in dehydration stress, we generated transgenic rose calli in which *RhCASPL1D1* was silenced by RNA interference (RNAi) or overexpressed (OX) through *Agrobacterium tumefaciens*-mediated transformation. In both cases, empty pSuper-GFP vector was used as the negative control. Successful genetic transformation was confirmed by the presence of GFP fluorescence under laser scanning confocal microscopy and RT-qPCR analysis of *RhCASPL1D1* expression (Fig. 3G and H). Significant differences in relative fresh weight were observed in *RhCASPL1D1*-RNAi and *RhCASPL1D1*-OX calli compared to the control only during the recovery phase at 12 h after rehydration (Fig. 3I). These results in petal discs and calli demonstrate that *RhCASPL1D1* plays a role mainly in the recovery phase after stress.

We also explored whether *RhCASPL1D1* plays a role in long-term water-deficient stress. To this end, we heterologously expressed *RhCASPL1D1* in the Arabidopsis wild-type (WT) Col-0 and *atcaspl1d2* mutant (SALK_204389) backgrounds as *CASPL1D2* is the close homologue of *RhCASPL1D1* (Fig. 1A). *RhCASPL1D1* expression in homozygous heterologous overexpression (OE) and complementation (Com) lines was confirmed by RT-qPCR (Fig. S4B). The seedlings were transferred to mannitol-containing ½-MS medium as simulated water-deficient treatment. After three weeks of mannitol treatment, the survival rate was significantly lower in *atcaspl1d2* mutant plants compared to Col-0. In contrast, two independent OE and Com transgenic lines exhibited enhanced drought tolerance compared to Col-0 (Fig. S6A and C). These findings suggest the potential role of *RhCASPL1D1* in the plant drought tolerance.

### RhCASPL1D1 physically interacts with RhPIP2 aquaporins

To explore the potential molecular mechanism underlying the role of RhCASPL1D1 in regulating flower dehydration tolerance, we identified RhCASPL1D1 interacting proteins. In Arabidopsis, AtPIP2;1 was identified as an AtCASPL1D2-interacting protein by immunoprecipitation–mass spectrometry (IP–MS) [31]. The important role of PIPs in maintaining water balance has been fully demonstrated, and bioinformatics analysis showed that *RhCASPL1D1* is an *AtCASPL1D2* homologue (Fig. 1). Therefore, we hypothesize that *RhCASPL1D1* may regulate the dehydration tolerance of cut rose flowers through interaction with RhPIP2 proteins. We identified six members of the PIP2 subfamily in the dataset of senescence- and dehydration-responsive genes in rose flowers, three of which had high basal expression levels (FPKM >50) in petals (*RhPIP2;1, RhPIP2;2* and *RhPIP2;7*; Fig. S5).

**Figure 4:**
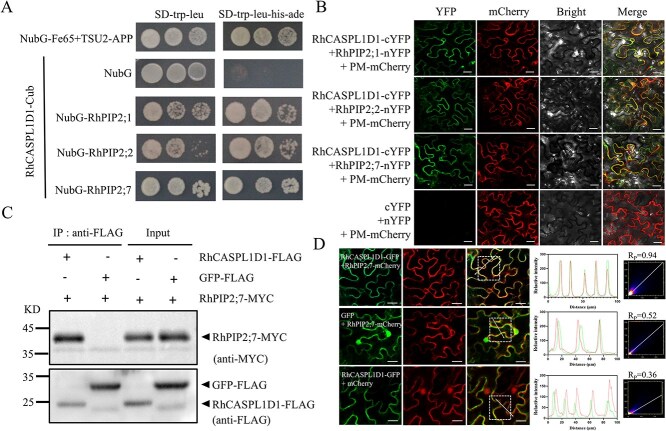
RhCASPL1D1 interacts with RhPIP2s. **A)** Split-ubiquitin yeast two-hybrid assay. RhCASPL1D1 was fused to the N terminus of ubiquitin (RhCASPL1D1-Cub), and RhPIP2;1, RhPIP2;2 as well as RhPIP2;7 were fused to the C terminus of mutated ubiquitin (NubG-RhPIP2;1/RhPIP2;2/RhPIP2;7). The TSU2-APP/NubG-Fe65 and RhCASPL1D1-Cub/NubG combinations were used as controls. The transformed yeasts were plated on Trp-Leu-His-Ade-lacking synthetic defined medium to test protein interactions. **B)** Interaction of RhCASPL1D1 and RhPIP2s in bimolecular fluorescence complementation (BiFC) assay. RhCASPL1D1-cYFP and RhPIP2;1/RhPIP2;2/RhPIP2;7-nYFP constructs were co-infiltrated in *N. benthamiana* leaves. PM-mCherry was used as a plasmalemma marker. The cYFP/nYFP combination was used as a negative control. YFP fluorescence was visualized with confocal microscopy after 3-day infiltration. Scale bars, 50 μm. **C)** RhCASPL1D1-RhPIP2;7 interaction detected by co-IP. The combinations of RhCASPL1D1-FLAG and RhPIP2;7-MYC constructions were co-transformed into *N. benthamiana* leaves with negative control, GFP-FLAG/RhPIP2;7-MYC combination. Immunoprecipitation was conducted by incubation of leaf total proteins and FLAG beads. The input and co-immunoprecipitated proteins were analyzed using anti-FLAG and anti-MYC antibodies. **D)** RhCASPL1D1 co-localizes with RhPIP2;7 in *N. benthamiana* epidermal cells. RhCASPL1D1-GFP and RhPIP2;7-mCherry were co-expressed in *N. benthamiana* leaves. GFP + RhPIP2;7-mCherry, RhCASPL1D1-GFP + mCherry were regarded as negative control, respectively. Fluorescence was visualized via confocal microscopy 3 days after infiltration. The fluorescence intensity profiles along the white arrows (in the merge micrographs) were analyzed by ImageJ. Pearson correlation coefficients (Rp) for co-localization were calculated from the fluorescence signal in the white dashed boxes using ImageJ. Scale bars, 50 μm.

To test the interaction between RhCASPL1D1 and RhPIP2s, we cloned three *RhPIP2s* ORF fragments and performed a split-ubiquitin yeast two-hybrid (SuY2H) assay. Yeast (*Saccharomyces cerevisiae*) containing RhPIP2;1, RhPIP2;2 or RhPIP2;7 grew well on SD/-Leu/-Trp/-His/-Ade selective medium (Fig. 4A). We then carried out a bimolecular fluorescence complementation (BiFC) assay to confirm the RhCASPL1D1–RhPIP2s interaction. Specifically, the N-terminal and the C-terminal segments of yellow fluorescent protein (YFP) were fused with RhPIP2 and RhCASPL1D1 proteins, respectively. Confocal microscopy revealed that RhCASPL1D1 interacts with RhPIP2;1, RhPIP2;2 and RhPIP2;7 (Fig. 4B). Furthermore, we verified the RhCASPL1D1–RhPIP2s interactions *in vivo* by co-immunoprecipitation (co-IP). We co-expressed RhCASPL1D1-FLAG and RhPIP2;7-MYC in *Nicotiana benthamiana* leaves and extracted the total protein after three days. The RhCASPL1D1-FLAG and GFP-FLAG proteins were immunoprecipitated using anti-FLAG beads and to probe whether they co-immunoprecipitated with RhPIP2;7-MYC using an anti-MYC antibody. As shown in Fig. 4C, RhPIP2;7-MYC only co-immunoprecipitated with RhCASPL1D1-FLAG. These results confirmed that RhCASPL1D1 physically interacts with RhPIP2s *in vivo* and *in vitro*. In addition to *RhCASPL1D1*, the two *RhCASP-like1* cluster members *RhCASPL1E1* and *RhCASPL1F2* are also induced by dehydration stress and expressed during flower opening (Fig. 1B). Therefore, we tested whether RhCASPL1E1 and RhCASPL1F2 could also interact with RhPIP2s. No interaction was found between RhCASPL1E1 or RhCASPL1F2 and RhPIP2s in the SuY2H assay (Fig. S6B).

RhCASPL1D1 was predicted to be PM-localized (Fig. S2C). To determine whether RhCASPL1D1 co-localized with RhPIP2s, we co-expressed *ProSuper: RhCASPL1D1-mCherry* and *ProSuper: RhPIP2;7-GFP* in *N. benthamiana* epidermal cells. The observation of laser scanning confocal microscopy showed that RhCASPL1D1-mCherry and RhPIP2;7-GFP fluorescence overlapped. Fluorescence intensity and correlation analyses revealed that RhCASPL1D1 highly colocalized with RhPIP2;7 with Pearson correlation coefficient (Rp) of 0.94 (Fig. 4D), supporting their physical interaction.

The phosphorylation state of PIPs regulates their channel gating, subcellular trafficking, as well as their interaction with target proteins [32]. We thus investigated whether PIP2 phosphorylation status influences the RhCASPL1D1–RhPIP2 interaction. Four phosphorylation sites have been identified in RhPIP2;7: Ser114, Ser273, Ser276, and Ser279 [33]. We generated phosphodeficient and phosphomimetic variants of RhPIP2;7 by mutating these four Ser residues to either Ala (RhPIP2;7^AAAA^) or Asp (RhPIP2;7^DDDD^), respectively (Fig. S6A). The MYTH system demonstrated that both RhPIP2;7^AAAA^ and RhPIP2;7^DDDD^ can interact with RhCASPL1D1, suggesting that the interaction between RhPIP2s and RhCASPL1D1 is independent of RhPIP2s phosphorylation status (Fig. S6C).

Protein interactions of PIP2s could potentially influence the water channel activity [34]. Therefore, we investigated the effect of RhCASPL1D1 on RhPIP2;7 water permeability in *Xenopus laevis* oocytes. No significant difference of permeability coefficient (P_ƒ_) is detected in oocytes co-expressing RhPIP2;7-FLAG and RhCASPL1D1-MYC compared with RhPIP2;7-FLAG and GFP-MYC control (Fig. S7). This indicates that the interaction of RhCASPL1D1 and RhPIP2;7 did not obviously change the PIP2;7 water channel activities.

### RhCASPL1D1 facilitates RhPIP2;7 stability and retention at the plasma membrane under dehydration stress

**Figure 5:**
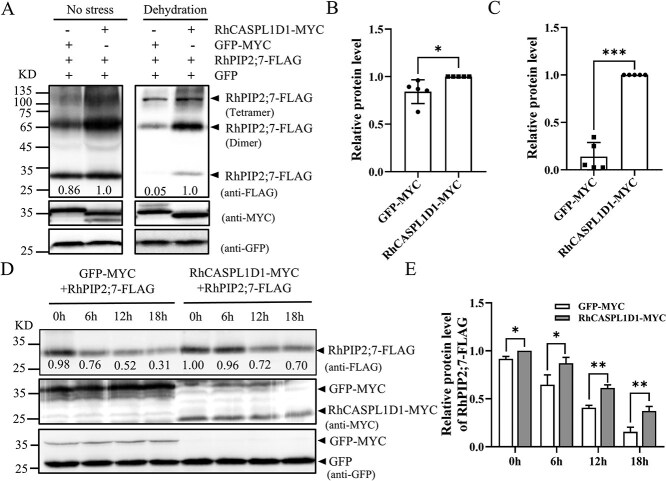
RhCASPL1D1 maintains RhPIP2;7 stability under dehydration stress. **A)** RhPIP2;7 protein levels in *N. benthamiana* under control conditions and dehydration stress. RhCASPL1D1-MYC and RhPIP2;7-FLAG were co-expressed in *N. benthamiana* leaves. The *agrobacterium* carrying pSuper-GFP vector was also co-injected to be used as internal standard Total protein was extracted three days after infiltration in the absence of stress (left panel) or after a 12-h dehydration treatment (PEG-8000, 30% [w/v]; right panel). RhPIP2;7 levels were detected with anti-FLAG, whereas RhCASPL1D1-MYC and GFP-MYC with anti-MYC. **B)** and **C)** Quantification of bands intensity in A) using ImageJ. RhPIP2;7-FLAG abundance under control conditions (no stress) B) and dehydration stress C). Relative protein abundance was determined by normalizing the band intensity to that of GFP-MYC (negative control). Values represent means ± SD (*n* = 3). **D)** RhCASPL1D1 inhibits RhPIP2;7 degradation *in vivo*. RhCASPL1D1-MYC and RhPIP2;7-FLAG were co-expressed in *Nicotiana benthamiana* with GFP-MYC and RhPIP2;7-FLAG as control. The pSuper-GFP vector was also co-injected to be used as internal standard. After being infiltrated with CHX, total proteins were extracted and immunoblotted using anti-FLAG, anti-MYC and anti-GFP antibodies. **E)** Relative protein level of RhPIP2;7-FLAG in D). Values represent means ± SD (*n* = 3). ^*^P < 0.05, ^**^P < 0.01 ^***^P < 0.001; Student’s *t*-test.

CASPs exhibit high stability in their membrane domain, showing all the characteristics of a membrane scaffold [26]. Therefore, we tested whether RhCASPL1D1 can scaffold and stabilize the RhPIP2 aquaporins. To this end, we co-expressed *35S:RhCASPL1D1-MYC* together with *35S:RhPIP2;7-FLAG* in *N. benthamiana* leaves. Under normal growth conditions, immunoblot analysis showed that RhPIP2;7 abundance was slightly higher in the presence of RhCASPL1D1-MYC (Fig. 5A, left panel). Notably, under dehydration stress, RhPIP2;7 abundance increased considerably when co-expressed with RhCASPL1D1-MYC (Fig. 5A, right panel). Relative protein levels of RhPIP2;7-FLAG were quantified by measuring the band intensity from immunoblot using ImageJ and normalized with GFP (Fig. 5B and C). These results above suggest that RhCASPL1D1 promotes RhPIP2;7 stability under dehydration stress.

To confirm the effect of RhCASPL1D1 on RhPIP2s stability *in vivo*, we performed a degradation assay by co-expressing different protein combinations in *N. benthamiana* leaves. In this assay, we co-expressed RhPIP2;7-FLAG and GFP-MYC as the negative control. Before total protein was extracted, *N. benthamiana* leaves were injected with 100 μM cycloheximide (CHX, a protein synthesis inhibitor) and incubated for different durations. The RhPIP2;7 level was assessed at different time points using an anti-FLAG antibody. RhPIP2;7-FLAG co-expressed with GFP-MYC was gradually degraded over the 12-h time course, but RhPIP2;7-FLAG co-expressed with RhCASPL1D1-MYC was degraded at a slower rate (Fig. 5D and E). This result indicates that RhCASPL1D1 can delay RhPIP2s degradation *in vivo*, which maintains RhPIP2s abundance at the PM.

PIPs show dynamic localization changes in response to environmental conditions. For example, rapid internalization of fluorescently tagged PIP2;7 was observed in Arabidopsis under osmotic stress. PIPs internalization can be clearly observed as the formation of spherical bodies and intracellular diffuse labeling [8, 10]. To explore whether RhCASPL1D1 affects the trafficking of RhPIP2 proteins, we generated transgenic rose hairy roots co-expressing RhPIP2;7-GFP with RhCASPL1D1-mCherry or mCherry by *Rhizobium rhizogenes*-mediated transformation [35].

**Figure 6:**
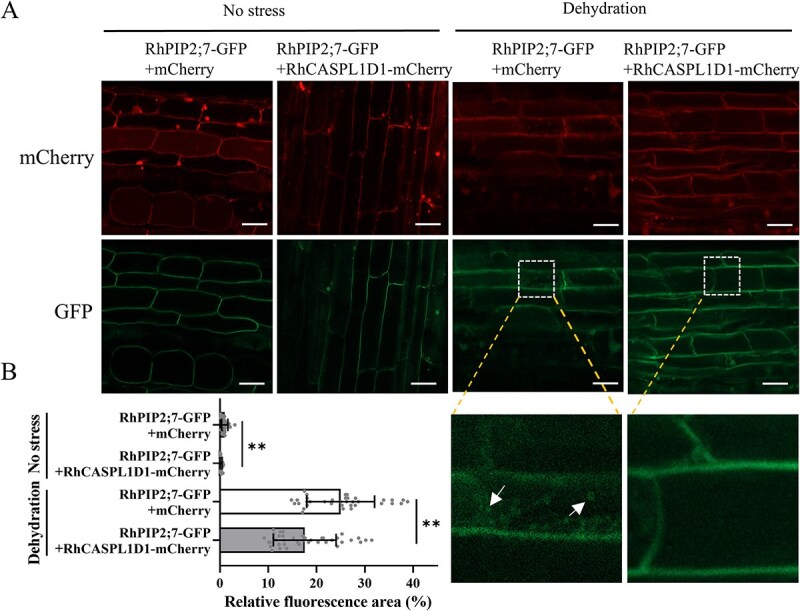
RhCASPL1D1 prevents RhPIP2;7 internalization in rose hairy roots. **A)** Transgenic rose hairy roots co-expressing PIP2;7-GFP and RhCASPL1D1-mCherry or mCherry were visualized by confocal microscope. Root images with or without 8-h dehydration (PEG-8000, 30% [w/v]) were taken. GFP and mCherry were excited at 488 and 587 nm, respectively. Boxed regions are enlarged in the below, and white arrows indicate the internalized PIP2;7. Scale bar, 50 μm. **B)** Intracellular relative fluorescence area quantification of RhPIP2;7-GFP from the confocal micrographs in A). Fluorescence area quantification was measured using ImageJ. The percentage values were ratio of intracellular GFP fluorescence area to the total area inside the cells. For each scatter plot the error bars indicate the mean ± SD. Significant differences are marked with asterisks according to Student’s *t*-test, ^**^ P < 0.01.

GFP fluorescence observed by confocal microscopy indicated that RhPIP2;7-GFP was mainly localized at the PM in the both types of transgenic root cells under normal conditions. To explore changes in RhPIP2;7-GFP localization under dehydration stress, 30% PEG 8000 (w/v) was applied to the transgenic roots. After 8-h dehydration, diffuse GFP fluorescence was observed in the root cells (Fig. 6A), implying that RhPIP2;7 was removed from the PM. Notably, we noticed that relative less GFP fluorescence accumulated inside the *RhCASPL1D1* overexpressed root cells (RhPIP2;7-nGFP +RhCASPL1D1-mCherry) compared with the control (RhPIP2;7-nGFP +mCherry), under dehydration stress. To compare the intracellular fluorescence intensity in the two types of transgenic root cells, we measured the fluorescence area fraction using ImageJ software. Under dehydration treatment, RhCASPL1D1 overexpressed root cells showed less intracellular fluorescent signal (about 17.0%) compared with average 25.9% intracellular fluorescence area in the control (Fig. 6B). The fluorescence area quantification results were consistent with the confocal micrographs, indicating that RhCASPL1D1 inhibits RhPIP2 degradation by promoting its retention in the PM.

Above all, we identified an ethylene-and dehydration-induced scaffold protein coding gene, *RhCASPL1D1,* which expression level also increased in rose flowers during rehydration. We found that RhCASPL1D1 mainly localizes to the PM and acts as scaffold for RhPIP2 proteins, including RhPIP2;1, RhPIP2;2, and RhPIP2;7. These interactions could enhance RhPIP2s’ stability under dehydration stress and facilitate PIP2s retention at the PM with independent of RhPIP2s phosphorylation status, thus enabling the rapid recovery of flowers from dehydration stress during rehydration processes.

## Discussion

### RhCASPL1D1 promotes flower recovery after water deficit stress

Plants slow their growth during periods of drought to ensure their survival. During the recovery period after the stress has passed, plants resume growth to complete their life cycle [1]. These plant resilience abilities are involved in complex regulatory mechanisms well reviewed by Yuan *et al.* [36], including metabolic homeostasis regulation, phytohormone signaling networks, transcription factor activation, as well as translational and post-translational protein modifications, etc. In the present study, we revealed that a CASP-*like* protein, RhCASPL1D1, promotes flower recovery after water deficit stress in *Rosa hybrida*.

CASPLs, a conserved class of transmembrane scaffold proteins, have been systematically characterized across plant species [19]. Our genome-wide analysis identified 37 RhCASPLs in *Rosa hybrida* (Fig. 1A), with particular focus on elucidating the functional role of RhCASPL1D1 in dehydration stress adaptation. Structural prediction revealed four transmembrane domains in RhCASPL1D1, consistent with its plasma membrane localization as demonstrated by subcellular localization assays (Figs S2 and 4D). Notably, the instability index of RhCASPL1D1 is 23.79, significantly below the critical threshold of 40 for stable scaffold proteins according to ProtParam program analysis (https://web.expasy.org/protparam/). These structural features align with the established role of CASPLs in maintaining membrane protein complex organization [20, 26], indicates the potentially scaffolding roles of RhCASPL1D1 to interact with binding partners in different signaling pathways.

Tissue-specific expression profiling revealed predominant *RhCASPL1D1* transcript accumulation in roots and floral tissues, with negligible expression in foliar organs (Fig. 2A). Intriguingly, temporal expression dynamics during dehydration–rehydration cycles showed strong correlation with endogenous ethylene fluctuations (Fig. 2C), mirroring ethylene-mediated stress responses previously reported in *Rosa* [37]. Ethylene induction was further confirmed through RT-qPCR analysis of ethylene treated petals, showing 4.5-fold upregulation (Fig. 2D). This regulatory pattern parallels the ethylene-responsive *CitCASPL1D1a* homolog in *Citrus sinensis* [38], suggesting an evolutionarily conserved ethylene-CASPL regulatory module in plant stress adaptation.

Emerging evidence from functional genomics studies demonstrates the pleiotropic roles of CASPLs in plant stress adaptation, encompassing pathogen resistance, cold tolerance, and drought acclimation [20–22]. Significantly, Arabidopsis double mutants *caspl1d1/caspl1d2* exhibited impaired root hydraulic conductivity under NaCl stress [23], implicating CASPL1D clade members in water transport and osmoregulatory processes. Functional validation in *Rosa hybrida* revealed that *RhCASPL1D1* knockdown lines showed compromised water-deficit recovery, with transgenic petal discs and calli failing to restore fresh weight postdehydration (Fig. 3C and I). Furthermore, silencing *RhCASPL1D1* induced delayed flower opening in *RhCASPL1D1*-silenced rose plantlets (Fig. 3D–F). Therefore, we demonstrated that the scaffold protein RhCASPL1D1 promotes water influx into cells, thereby enhancing rose flowers recovery during rehydration after water deficient.

### RhCASPL1D1 scaffolds and stabilizes RhPIP2 aquaporins

PIPs are intrinsic membrane protein channels that control water movement across the PM to maintain cellular water homeostasis. Numerous studies have reported that PIPs are regulated in abiotic stress responses, especially concerning of water-deficit stress [6, 10]. PIP2s stability and activity during abiotic stress are precisely regulated by various mechanisms. PIP2 proteins are down-regulated by 26S proteasome and microautophagy-mediated degradation under drought stresses [7, 8]. There are also increasing evidences indicating that PIP proteins degradation was delayed under abiotic stress [11, 39]. Scaffold proteins usually provide modular regulation of rapid mobilization of signaling components within a cell subjected to external stresses [15]. In this study, we identified the scaffold protein RhCASPL1D1 that interacts with RhPIP2s and maintains aquaporin’s levels in rose flowers (Fig. 4). Functional expression of RhCASPL1D1 and RhPIP2;7 in *Xenopus laevis* oocytes does not obviously change the PIP2 water channel activities (Fig. S7). This phenomenon is consistent with that CASPL1D1 has no functional effects on PIP2;1 in Arabidopsis [23]. RhPIP2;7 protein levels are enhanced when co-expressed with RhCASPL1D1 in *N. benthamiana*, especially under dehydration stress (Fig. 5A and C). The degradation of RhPIP2;7 could be inhibited by RhCASPL1D1 inhibits *in vivo* (Fig. 5D and E). These results suggest that RhCASPL1D1 promotes RhPIP2;7 stability under dehydration stress.

The dynamic regulation of PIP2s intracellular trafficking is potentially important for cells to modulate membrane water permeability responsive to abiotic stresses. For instance, salinity-induced H_2_O_2_ triggers the internalization of PM-localized aquaporins [10]. In *Mesembryanthemum crystallinum*, a transcription-independent increase in PIPs abundance at the PM is an adaptative process to osmotic stress [11]. In rose flowers, we found that RhCASPL1D1 inhibits RhPIP2s internalization by promoting its retention in the PM (Fig. 6). Scaffold proteins directly interact with and stabilize client proteins. They may do so by inhibiting client protein ubiquitination and subsequent proteasomal degradation [40, 41]. Correspondingly, RhCASPL1D1 may inhibit RhPIP2s degradation by preventing their interaction with ubiquitination- or autophagy-associated proteins. Moreover, CASP-driven microdomains formed at the PM may also promote RhPIP2s retention [17]. Additional experiments are required to test these hypotheses. Taken together, our results reveal that scaffolding protein RhCASPL1D1 interacts with PIP proteins and mediates the increases in PM-localized RhPIP2 abundance to enable the rapid recovery of rose flowers after dehydration stress.

## Materials and methods

### Plant materials and growth conditions

The plantlets and cut flowers of *Rosa hybrida* cv. Samantha were used in this study. Rose plantlets were propagated and grown as described previously [33]. Rooted plants were grown hydroponically for one week before being transplanted into pots (9 cm in diameter) containing a mixture of peat moss and vermiculite (v/v = 1:1). Plants were grown in greenhouse at 22 ± 1°C, with ~50% relative humidity and 100–120 μmol m^−2^ s^−1^ light intensities with 8-h dark/16-h light cycle. Cut flowers were harvested and pre-treated according to Liu *et al*. [25]. Petal discs 1 cm in diameter collected from stage 2 cut flowers were used for the VIGS assay. The opening stages of flowers were defined according to the previous description [29]. *Nicotiana benthamiana* for gene transient expression assays were grown in pots in growth chambers with the same conditions as mentioned above.

### Bioinformatics analysis

For genome-wide identification of *CASP*-*like* family genes from rose (*Rosa hybrida*), 39 CASPL proteins sequence in *A. thaliana* were download from TAIR website (https://www.arabidopsis.org), the rose protein sequence file was download from the genome website (https://lipm-browsers.toulouse.inra.fr/pub/RchiOBHm-V2). The amino acid sequences of CASPLs in Arabidopsis were aligned with rose protein data to obtain homologs sequences in rose using BLASTP [42]. RhCASPL protein motifs were analyzed by using HMMER web server [27].

The phylogenetic tree of CASPL proteins in *Rosa hybrida* and *A. thaliana* was reconstructed with MEGA11 [28]. We used ProtTest to predict the optimal model, and operated the maximum likelihood method with setting 1000 bootstrap replicates. The output image was embellished with ITOL online tool (https://itol.embl.de/login.cgi). Genes distribution on chromosomes, protein pairwise similarity matrix and gene expression heatmap were generated with software TBtools (v2.154) [43], by using the FPKM values from flower dehydration and senescence transcriptome in *Rosa hybrida*. Amino acid sequence alignment both were analyzed by Clustal X (2.1) software [44].

### Virus-induced gene silencing assay

Silencing of *RhCASPL1D1* by virus-induced gene silencing (VIGS) was carried out following previous method [45]. To construct the pTRV2-*RhCASPL1D1* plasmid, a 349-bp fragment of *RhCASPL1D1* was cloned into the pTRV2 vector. The pTRV1 and pTRV2 empty vectors, as well as pTRV2-*RhCASPL1D1* constructs were individually transformed into *A. tumefaciens* strain GV3101. The overnight cultured bacterial was collected and resuspended in infiltration buffer (10 mM MgCl_2_, 200 mM acetosyringone [AS], 10 mM MES, pH = 5.6, OD_600_ = 1.5). Cell suspension of pTRV1 was mixed with pTRV2 and pTRV2-*RhCASPL1D1* in equal proportions (v/v), respectively. Rose plantlets or petal discs submerged in resuspended mixtures were exposed to a −0.08 MPa vacuum for 5 min twice. The infiltrated petal discs and plantlets were cultured at 8°C in dark conditions for three days. The plantlets were transplanted in pots, and flowers were subjected for further analysis.

### Generation of transgenic rose calli

The rose calli was inducted and cultured according to Jiang *et al.* [46] with some modifications. The mediums and components used are as follows: induction medium (4.4 g L^−1^ MS, 3.0 mg L^−1^ 2,4-dichlorophenoxyacetic acid, 0.05 mg L^−1^ kinetin, 45 g L^−1^ glucose, and 2.5 g L^−1^ phytagel, pH = 5.9), propagation medium (4.4 g L^−1^ MS, 1.0 mg L^−1^ 2,4-dichlorophenoxyacetic acid, 0.05 mg L^−1^ 6-benzylaminopurine, 45 g L^−1^ glucose, and 2.5 g L^−1^ phytagel, pH = 5.9). Around 1 cm calli of grown in the propagation medium was used for genetic transformation.

Transgenic rose calli was generated as previously described [46]. In brief, the full-length sequence of *RhCASPL1D1-GFP* was inserted into the pSuper and pTCK303 (the *GUS* encoding sequence was replaced with *GFP*) vectors, to generate the overexpressing and RNA interference construct, respectively. Plasmids of pSuper-GFP empty vector and constructs were transformed into *A. tumefaciens* strain EHA105. Rose calli was genetically transformed by Agrobacterium-mediated transformation, the specific experimental operation is described in the reference. All primers used are listed in the Table S3.

### Dehydration treatment of rose petal discs and calli

Rose VIGS-silenced petal discs and transgenic calli were dehydrated as previously described [25]. After infiltration, petal discs were recovered at 25°C for 12 h. The VIGS-silenced petal discs and overexpressed calli were placed in empty petri dishes to lose water to 50% and 70% of the original weight at controlled conditions of 22 ± 1°C, with ~50% relative humidity. Dehydrated petal discs and calli were subjected to distilled water or medium with 3% agar for rehydration. The fresh weight was measured at different time points. RNA was extracted from petal discs and calli after 12-h rehydrated to detect target gene expressions.

### Arabidopsis transgenic

Arabidopsis (*Arabidopsis thaliana*) accession Columbia (Col-0) was used as the wild type. The T-DNA insertion mutant *Atcaspl1d2* (SALK_204389) was obtained from AraShare (https://www.arashare.cn). To generate the heterologous overexpression and complementation lines, the *Pro35S:RhCASPL1D1-GFP* construct was introduced into *Agrobacterium tumefaciens* strain GV3101. Arabidopsis wild type and *atcaspl1d2* were individually transformed via the floral dip method [47].

### Arabidopsis water-deficient mimetic treatment

Seeds from different homozygotic Arabidopsis lines were surface sterilized by 1% NaClO for 10 min. The sterile seeds were sown on ½-MS medium and incubated at 4°C for 3 days to break dormancy. Mannitol was used to mimic water deficient stress. Seven-day-old seedlings germinated on ½-MS medium were transferred to ½-MS medium (control) or ½-MS medium supplemented with 100 or 200 mM mannitol. Seedlings were grown vertically for three weeks on these medium, after which the seedlings were photographed and survival rates were counted, the experiment was repeated three times.

### Water permeability assay in *Xenopus laevis* oocytes

The coding sequences of *RhCASPL1D1-MYC*, *RhPIP2;7-FLAG* and *GFP-MYC* were cloned into PT7Ts expression vector, respectively. The complementary RNAs (cRNAs) were produced using the mMESSAGE mMACHINETM^T7^ system (Promega, Madison, WI, USA). Heterogeneous gene expression and water permeability assay in *Xenopus laevis* oocytes were performed following previous procedures [48]. In brief, *Xenopus laevis* oocytes were microinjected with 20 nL cRNA (1 μg/μL,) and subsequently incubated in ND96 medium (96 mM NaCl, 2 mM KCl, 1.8 mM CaCl₂, 1 mM MgCl₂, 5 mM HEPES, pH 7.4) supplemented with 0.1 mg·ml^−1^ gentamicin and streptomycin at 17°C for 3 days. Then, the transformed oocytes were transferred into hypotonic solution (1/5 ND96 medium) for water permeability assay. Cell volume changes were monitored real-time using a stereomicroscope (Olympus, SZ680) and the permeability coefficient (P_ƒ_) was calculated.

### Split-ubiquitin yeast two-hybrid assay

The interactions between the membrane protein RhCASPL1D1 and RhPIP2s were tested in yeast (*Saccharomyces cerevisiae*) using a split-ubiquitin yeast two-hybrid (SuY2H) system according to previous description [33]. pPR3N and pBT3-STE were used as the prey and bait vectors, respectively. The full-length sequences of *RhPIP2;1, RhPIP2;2,* and *RhPIP2;7* were cloned into pPR3N and fused with *NubG* to express NubG-RhPIP2s, respectively. The full-length *RhCASPL1D1* was fused with *Cub* in pBT3-STE to express RhCASPL1D1-Cub. Yeast strain NMY51 was used to co-transform with constructs above. Three days after transformation, a series of diluted (10×, 100×, and 1000×) co-transformed NMY51 cells were spotted onto Trp- and Leu-lacking SD medium or Trp-, Leu-, His-, and Ade-lacking SD medium, and cultured at 30°C to test protein interactions.

### Bimolecular fluorescence complementation assay

The coding sequences of *RhCASPL1D1* and *RhPIP2s* without stop codons were individually cloned into the binary plasmids pSPYNE and pSPYCE to construct *RhCASPL1D1-YNE* and *RhPIP2s-YCE* vectors. A pair of bimolecular fluorescence complementation assay (BiFC) constructs alongside with the pair of empty vectors were introduced into the *A. tumefaciens* strain GV3101. *N. benthamiana* leaf was infiltrated by vacuum injection (OD_600_ was adjusted to 1.2). Fluorescence signal was observed using confocal microscopy (Zeiss LSM 800). YFP fluorescence was excited at 488 nm wavelength and observed with 490- to 560-nm emission filter. The mCherry fluorescence was excited at 587 nm wavelength and observed with 595- to 650-nm emission filter.

### Co-immunoprecipitation assay

The assays were performed as previously reported [41]. Briefly, total protein was extracted from *N. benthamiana* leaves co-transformed with *RhPIP2;7-MYC* and *RhCASPL1D1-FLAG* using IP buffer (1× PBS [8 mM Na_2_HPO_4_, 2.68 mM KCl, 140 mM NaCl, 2 mM KH_2_PO_4_, pH 7.45], 0.01% Triton X-100, 0.01% CHAPS, 0.05 mM DTT, and protease inhibitor cocktail). The supernatants were precipitated with prewashed FLAG beads at 4°C for 3 h, then the beads were washed with a buffer (TBS; 50 mM Tris–HCl; 150 mM NaCl). The immunoprecipitated protein was boiled with 2× SDS loading buffer. Anti-FLAG or anti-MYC antibodies were used for western blot analysis.

### Subcellular co-localization

The subcellular co-localization of RhCASPL1D1 and RhPIP2;7 was determined in *N. benthamiana* epidermal cells. The ORF sequences of *RhCASPL1D1* and *RhPIP2;7* were, respectively, inserted into the pSuper1300 to result in *35S: RhCASPL1D1*-*GFP* and *35S: RhPIP2;7-mCherry* plasmids. Both constructs were co-infiltrated into *N. benthamiana* epidermal cells. Two days after infiltration, the co-localization of GFP and mCherry was observed with a laser confocal microscopy (Zeiss LSM 800). The fluorescence intensity and the Pearson's co-localization coefficients were calculated using ImageJ software.

### Western blot and protein degradation assay

To determine the protein abundance and degradation *in vivo*, *N. benthamiana* leaves were co-injected with agrobacterium strain GV3101 carrying pSuper-*RhCASPL1D1-MYC* and pSuper-*RhPIP2;7-FLAG* alongside agrobacterium carrying pSuper*-GFP-MYC* and pSuper-*RhPIP2;7-FLAG*, respectively. pSuper*-GFP* vector was used as the infiltration control. After 48 h of infiltration, total proteins were extracted for protein abundance detection, or leaves were infiltrated with 100 μM cycloheximide (CHX) for various durations to detect protein degradation. Protein immunoblotting was performed according to Chen *et al.* [7]. Proteins were separated on 10% (v/v) sodium dodecyl-sulfate polyacrylamide gel electrophoresis (SDS-PAGE) gel, and probed with anti-MYC, anti-FLAG, and anti-GFP (Sigma), respectively.

### Rose transgenic hairy root induction

Transgenic rose hairy roots were generated following the reported method [35, 49], with a few modifications. pSuper-*RhCASPL1D1-mCherry*, pSuper-*RhPIP2;7-GFP* and pSuper-*mCherry* plasmids were transformed into *Rhizobium rhizogenes* strain K599. Culture the bacterial solution overnight and centrifuge, the precipitate was resuspended in infiltration buffer (10 mM MgCl_2_, 10 mM MES, 200 mM AS, pH = 5.6, OD_600_ = 1.5). Mixtures of cell suspensions containing pSuper*-mCherry* and pSuper-*RhPIP2;7-GFP*, pSuper-*RhCASPL1D1-mCherry* and pSuper-*RhPIP2;7-GFP* (v/v = 1/1) were used for infiltration. The base of the root-removed rose plantlets was submerged into mixed infiltration buffer for 30 min, infiltrated plants were transplanted to pots until they develop 5–10-cm hairy roots.

## Supplementary Material

Web_Material_uhaf119

## Data Availability

The raw data of RNA-seq used in the study were deposited into the National Center for Biotechnology Information (NCBI) database at Sequence Read Archive (SRA) with the accession number PRJNA1250776 and PRJNA1250784.
