# Supplementary material for: Scaffold protein RhCASPL1D1 stabilizes RhPIP2 aquaporins and promotes flower recovery after dehydration in rose (Rosa hybrida)
Source: Hortic Res. 2025 Apr 30;12(8):uhaf119. doi: 10.1093/hr/uhaf119 (PMC12258145; doi:10.1093/hr/uhaf119)
Supplement: Web_Material_uhaf119 [file web_material_uhaf119.zip › Revised supplementary Material.docx]

## Supplementary figures

**
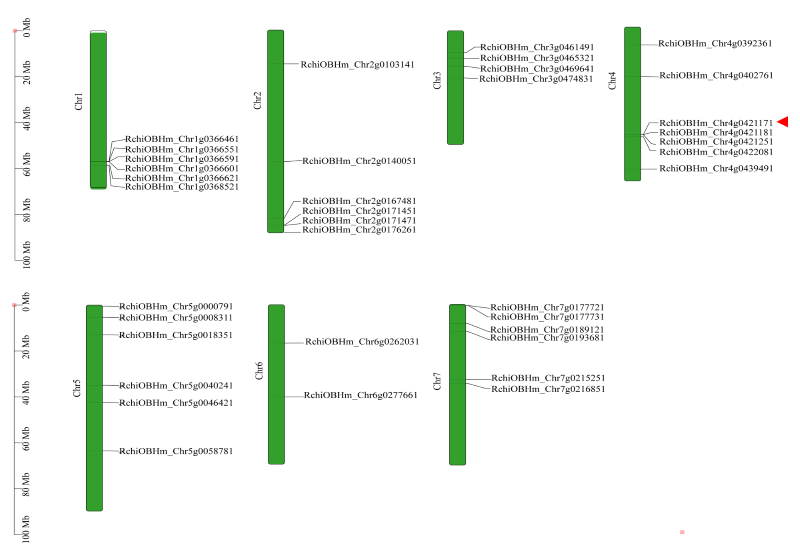
Figure S1.** **Chromosomal distribution of the *RhCASP-like* family members in *Rosa chinensis* ‘Old Blush’.** The left scales represent chromosome length, and the arrowhead indicates the *RhCASPL1D1*.


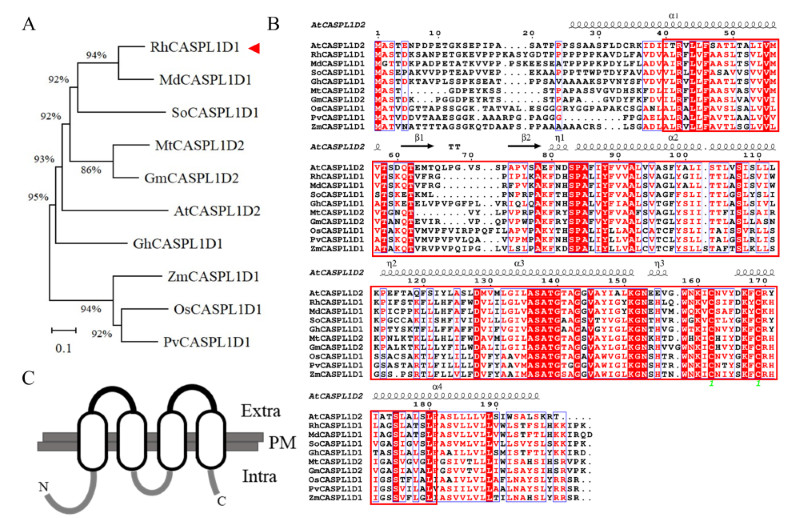


**Figure S2. RhCASPL1D1 homologs sequences analysis and RhCASPL1D1 protein structure.** **A)** Phylogenetic analysis of RhCASPL1D1 and its homologs in nine species. The neighbor-joining phylogenetic tree was constructed using MEGA11 with 1000 bootstrap replicates. The scale bar indicates evolutionary distances. RhCASPL1D1 was indicated by a red arrowhead. **B)** Amino acid sequence alignment of CASPL1D1 proteins from various species. The red outline indicates the conserved DUF588 domain. **C)** Protein structure of CASPL1D1. Protein topology was predicted using an online program (https://www.uniprot.org/). The four oval shapes represent transmembrane helixes, the black and gray curved lines indicate extracellular and cytoplasmic regions, respectively. Accession numbers of proteins are as follows: RhCASPL1D1 (XP_024197231), AtCASPL1D2 (NP_187290), MdCASPL1D1 (XP_008390731), SoCASPL1D1 (XP_004229219), GhCASPL1D1 ([XP_016739377](https://www.ncbi.nlm.nih.gov/protein/XP_016739377.2?report=genbank&log$=protalign&blast_rank=1&RID=P3J459CP01N)), MtCASPL1D2 (XP_003609582), Gm CASPL1D2 ([NP_001238662](https://www.ncbi.nlm.nih.gov/protein/NP_001238662.1?report=genbank&log$=protalign&blast_rank=2&RID=P3CMNCNF013)), OsCASPL1D1 (NP_001409051), PvCASPL1D1 (XP_039804867), ZmCASPL1D1 ([NP_001384438](https://www.ncbi.nlm.nih.gov/protein/NP_001384438.1?report=genbank&log$=protalign&blast_rank=2&RID=P3BJ7WFB016)).


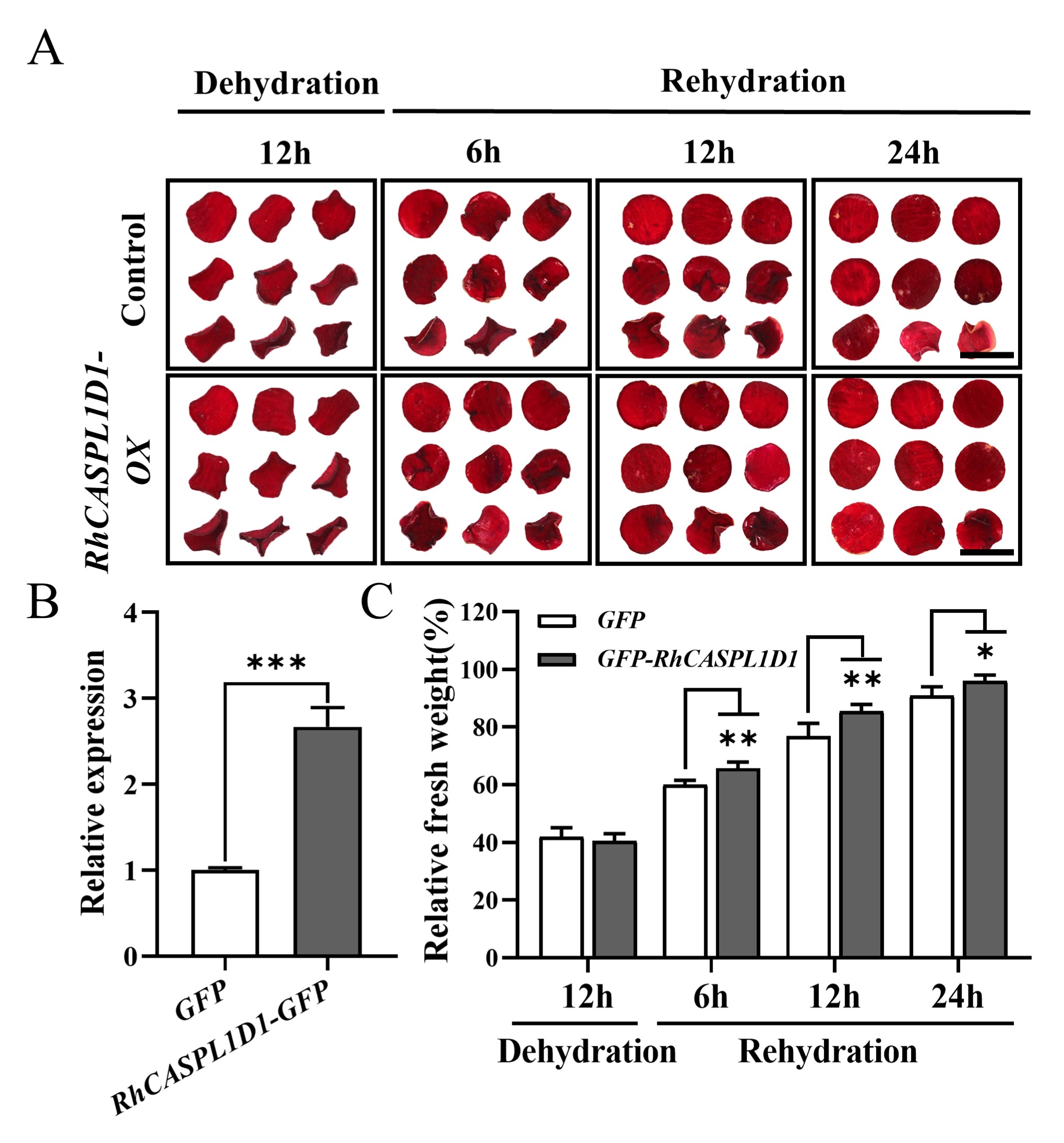


**Figure S3**. ***RhCASPL1D1* overexpression in petal discs promotes petal recovery after dehydration. A)** Phenotypes of *RhCASPL1D1* overexpressed (*RhCASPL1D1-GFP*) rose petal discs in response to dehydration and rehydration. The empty vector was used as the control. Images were taken at the indicated time intervals. Scale bars, 1 cm. **B)** RT–qPCR analysis of *RhCASPL1D1* expression in the petal discs shown in **A)**. *RhUBI1* was used as the internal control. Data are means of three biological replicates ± SD. *** P<0.001. **C)** Relative fresh weight of petal discs. Discs weight was quantified at the indicated time points. Data are means ± SD, n=5. Asterisks indicate statistically significant differences *(*P* < 0.05, ***P* < 0.01; Student’s t-test).

**
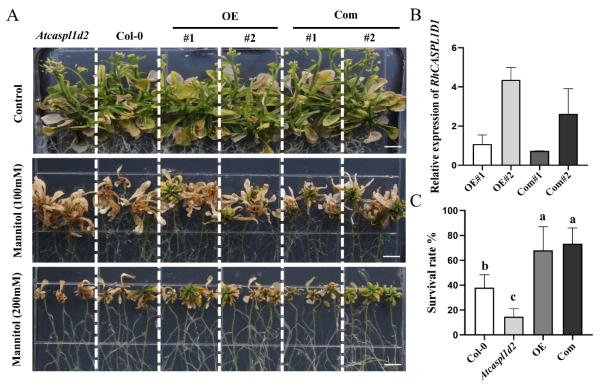
**

**Figure S4.** ***RhCASPL1D1* promotes drought stress tolerance in Arabidopsis.** Drought stress tolerance phenotypes **A)**, *RhCASPL1D1* relative expression **B)**, and survival rates **C)** of Col-0 and the *Atcaspl1d2* mutant harboring a *RhCASPL1D1* overexpression construct (*Pro35S:RhCASPL1D1*; generating the OE and Com lines, respectively). Transgenic lines were grown vertically on plates containing ½-MS (Control) or ½-MS with mannitol (100 and 200 mM) for 21 d prior to phenotypic analysis and RT-qPCR. Scale bars, 1 cm. Each value is the mean of three replicates ± SD.


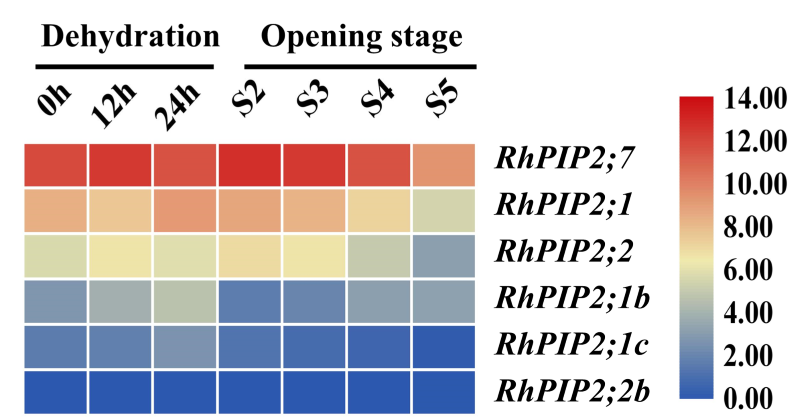


**Figure S5. Transcript abundance profile of *RhPIP2* family genes during flower opening and dehydration.** Expression levels in the heatmap are log_2_-based FPKM. S2-S5, flowers at opening stage 2 to stage 5. Accession numbers of the proteins are as follows: RhPIP2;1 (XP_024176646), RhPIP2;2 (XP_024158481), RhPIP2;7 (XP_024166688), RhPIP2;1b (XP_024188526), RhPIP2;1c (XP_024191054), RhPIP2;2b (PRQ47345).

**
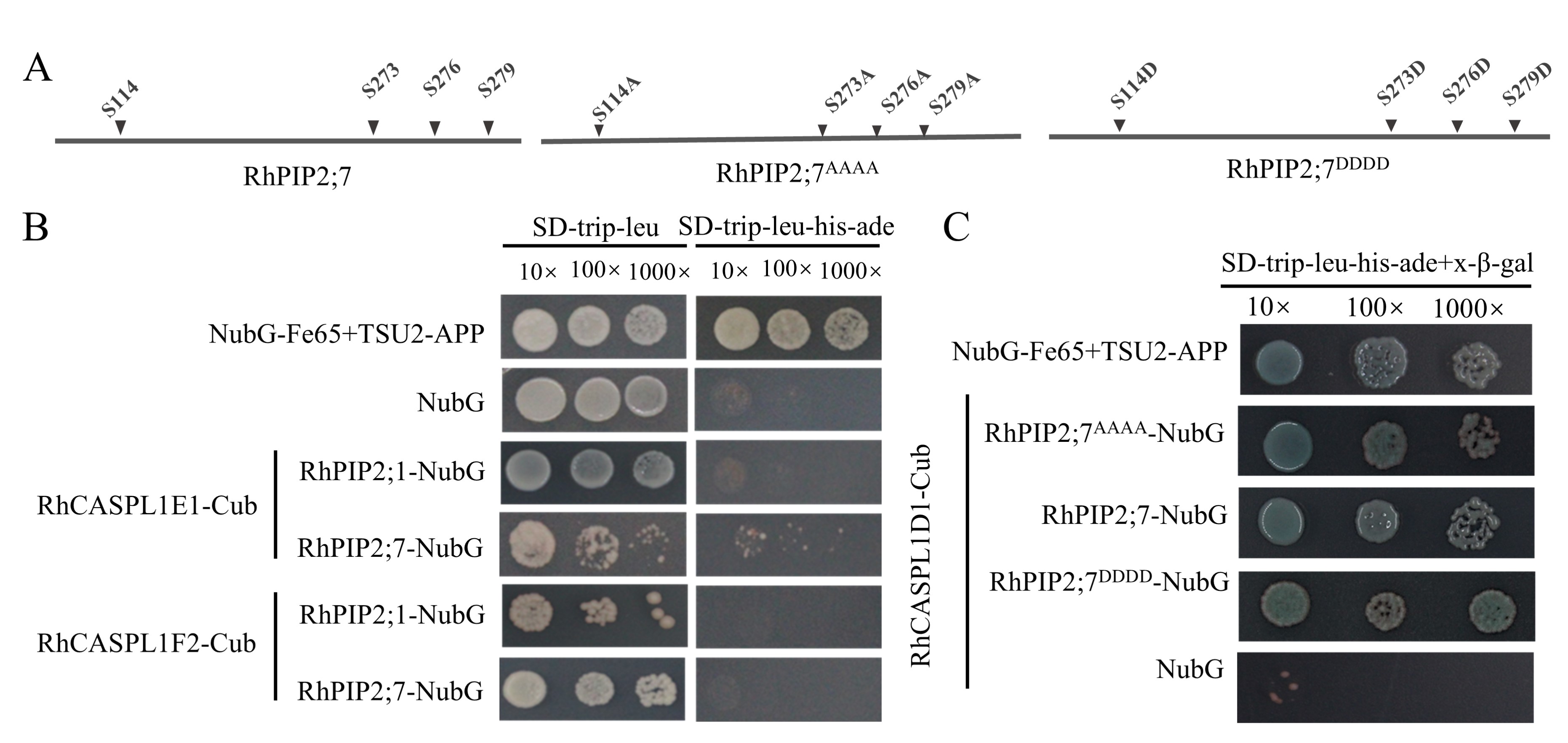
**

**Figure S6**. **The RhCASPL1D1–RhPIP2;7 interaction is independent of RhPIP2;7 phosphorylation status.** **A)** Schematic diagram of RhPIP2;7 phosphorylation sites. To generate the phosphodeficient form of RhPIP2;7 (RhPIP2;7^AAAA^), Ser 114, Ser273, Ser276 and Ser279 were mutated to Ala. To generate the phosphomimetic form of RhPIP2;7 (RhPIP2;7^DDDD^), Ser 114, Ser273, Ser276 and Ser279 were mutated to Asp. **B)** RhCASPL1D1 homologs do not interact with RhPIP2s. For the SuY2H assay, the TSU2-APP + NubG-Fe65 combination was used as the positive control, and the RhCASPL1D1-Cub + NubG combination was used as the negative control. **C)** Interactions between the RhPIP2;7 phosphorylation mutants (RhPIP2;7^AAAA^ and RhPIP2;7^DDDD^) and RhCASPL1D1 were tested in a split-ubiquitin yeast two-hybrid assay.


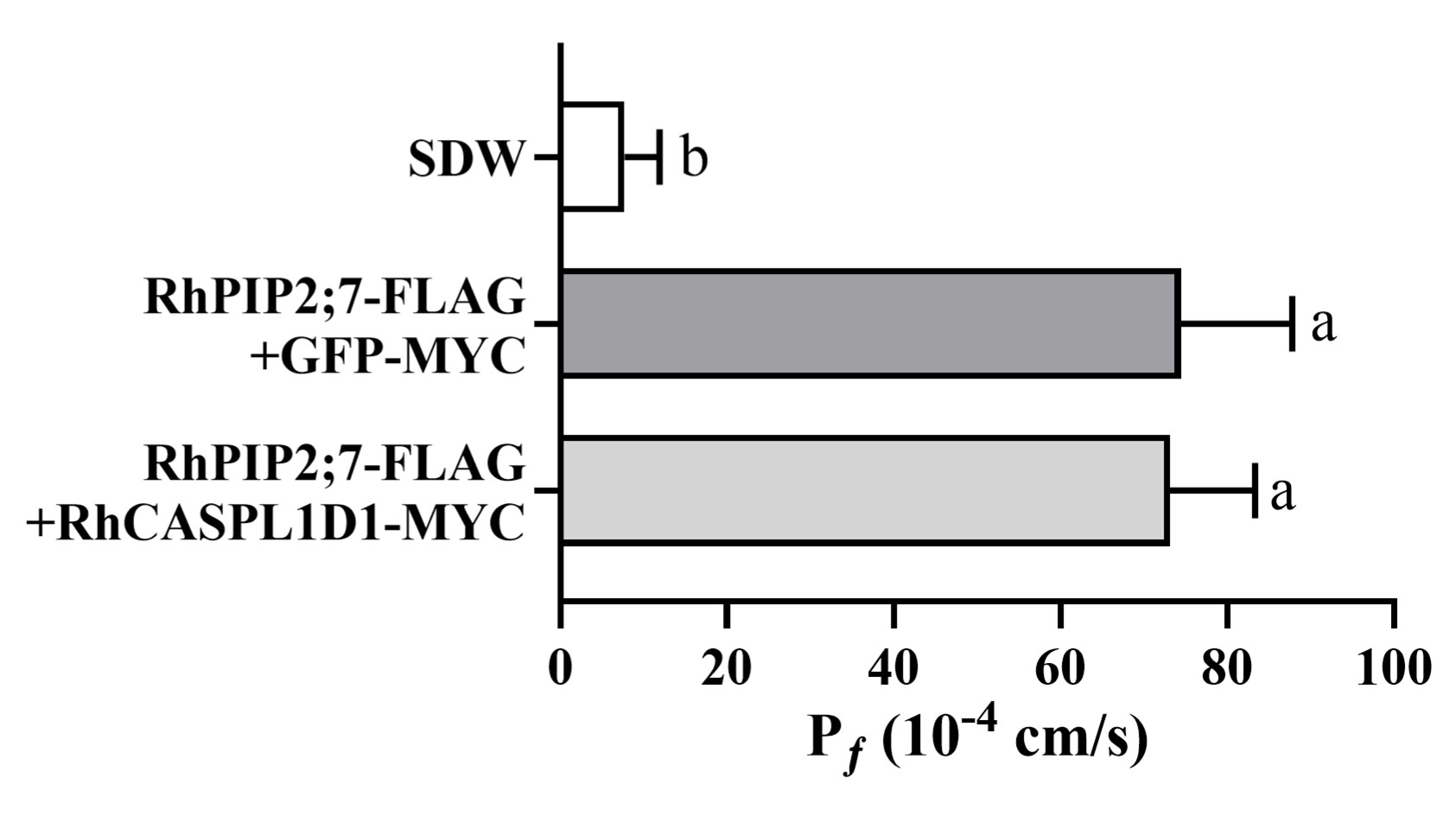


**Figure** **S7.** **Water permeability coefficient (P_ƒ_) analysis of *Xenopus laevis* oocytes expressing *RhCASPL1D1* and *RhPIP2;7*.** cRNAs encoding RhCASPL1D1-MYC (10 ng) and RhPIP2;7-FLAG (10 ng) was injected into oocytes. GFP-MYC cRNA (10 ng) and SDW was used for negative control. After 3 days of culture, oocytes were transferred to 1/5 ND96 medium, swelling of cells was photographed and calculated the permeability coefficient (P_ƒ_). Values are means ± SD (n = 20-30). Lowercase letters indicate significant differences according to Duncan’s multiple range test *(p*<0.05).
